# Giant Adult Mesenteric Lipoma: A Rare Cause of Chronic Abdominal Distention and Discomfort

**DOI:** 10.1155/2020/6010757

**Published:** 2020-02-25

**Authors:** Houssam Khodor Abtar, Fahed Kheireddine Abdallah, Rayan Said Lakkis, Jad Jamal Terro, Nathalie Haidar Ismail, Mohammad Ahmad Al Raishouni, Houssein Haidar Ahmad, Kassem Mohammad Jammoul

**Affiliations:** ^1^Department of Surgery, Central Military Hospital, Beirut, Lebanon; ^2^Hopital Delafontaine, Saint-Denis, Paris, France; ^3^FAHS Surgical Services, Michigan, USA; ^4^Department of Surgery, Saint George Hospital, Beirut, Lebanon

## Abstract

Solitary or multiple lipomas are considered common tumors that can occur anywhere in the body; however, mesenteric lipoma is a rare entity that is well known to present with signs and symptoms of small bowel volvulus. Hereby, we present a case of a 54-year-old male patient with multiple comorbidities who was suffering from chronic abdominal discomfort and gradual increase of his abdominal distention over many years without seeking any medical attention. The patient was seen by a general practitioner after complaining of an inflated abdomen, as he described his condition. After several imaging studies, he was diagnosed with one of the largest mesenteric lipomas in the literature. Mesenteric lipoma should be present in the differential diagnosis of any abdominal tumor. Magnetic resonance imaging plays a major role in differentiating benign from malignant lipomas.

## 1. Introduction

Lipomas are formed from adipose cells that lead to the creation of an encapsulated soft tissue mass. It is mainly benign in nature and can be seen mostly in the cephalic part of the body [1]. Mesenteric lipoma is a rare tumor that can grow to considerable size until it causes symptoms of obstruction or volvulus such as abdominal pain and discomfort when the patient is diagnosed with or can be found incidentally [2]. This case highlights the fact that gradual abdominal distention can be the initial presenting symptom of a giant mesenteric lipoma.

## 2. Case Presentation

**Figure 1:**
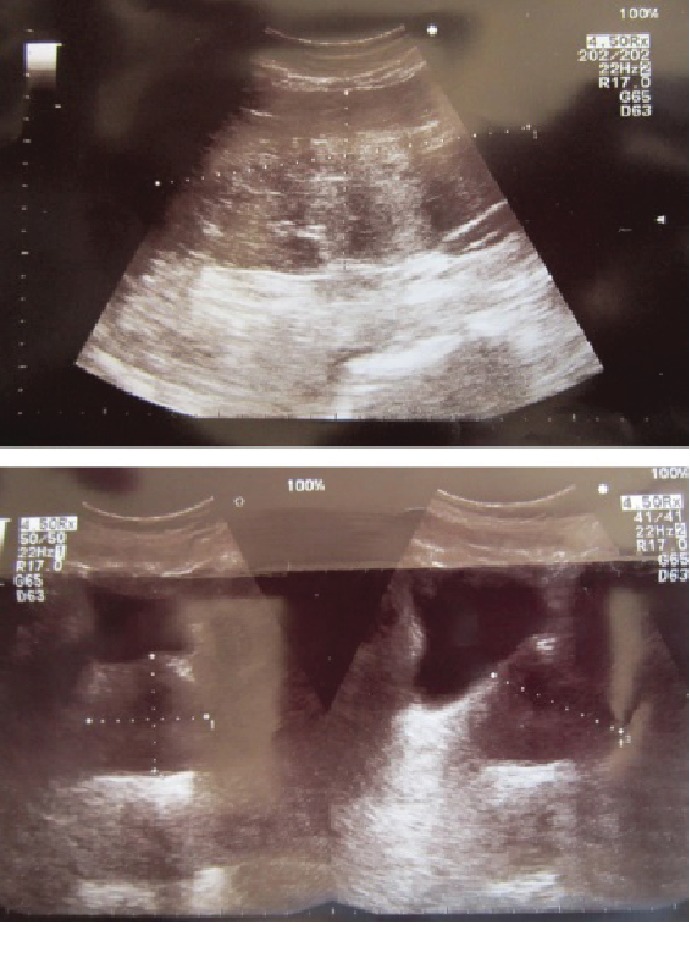
Ultrasound of abdomen showing big hyperechoic intra-abdominal mass.

**Figure 2:**
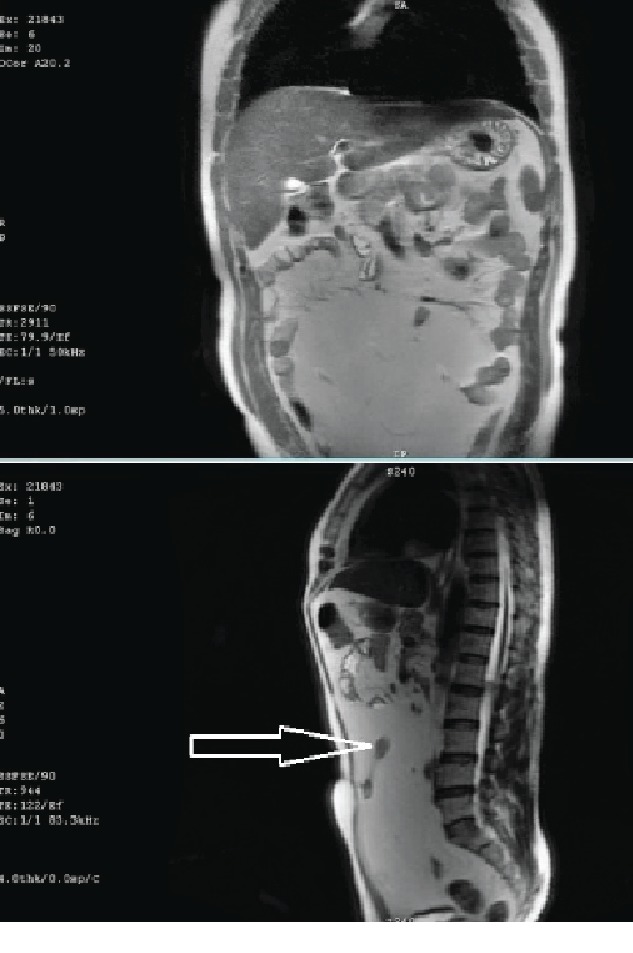
Abdominal and pelvic MRI detecting mesenteric lipomatous tumor encroaching the intestinal loop (white arrow).

**Figure 3:**
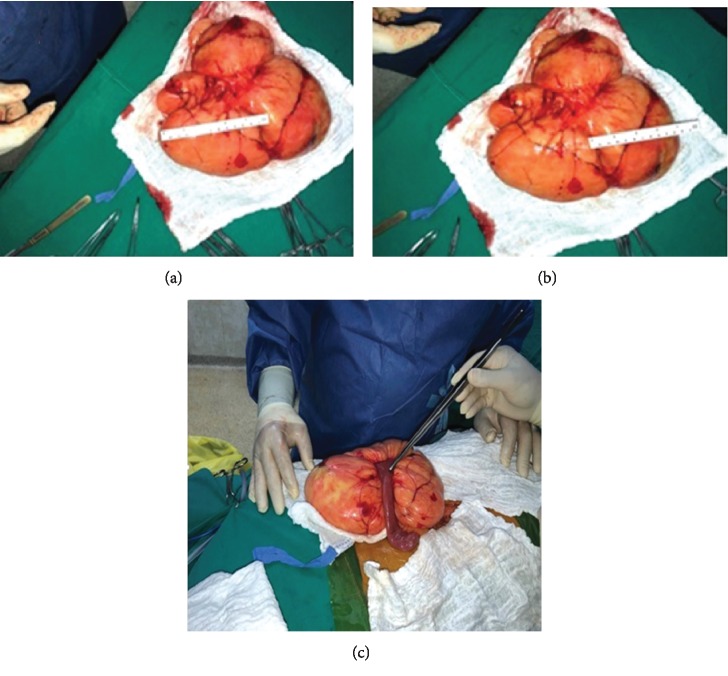
En bloc resection of the 25.0 × 23.0 × 5.0 cm mesenteric lipoma (a, b). Mesenteric lipoma encompassing an ileal loop through it (c).

We present a case of a 54-year-old male patient with a medical history of hypertension, dyslipidemia, benign prostatic hyperplasia (BPH), and right testicular cancer diagnosed and managed by orchiectomy followed by radiation therapy about 23 years ago, with complete remission. The patient complained of chronic abdominal distention without any signs of obstruction associated with abdominal discomfort and reflux. For many years before the presentation, he was taking antispasmodics, proton pump inhibitors (PPIs), and laxatives for symptomatic relief. Upon examination by a general practitioner, an ultrasound of the abdomen and laboratory tests were done for further evaluation. The ultrasound revealed a huge lobulated intra-abdominal mass over the periumbilical region (Figure 1); however, his labs were within normal limits. Following these impressions, the patient was transferred to a general surgeon for further evaluation. Magnetic resonance imaging (MRI) was ordered and showed the presence of well-encapsulated fat containing a midabdominal tumor surrounding a loop of the small bowel without small bowel dilatation (Figure 2). Lipoma or liposarcoma was on the top of the differential diagnosis. Exploratory laparotomy was performed, and a giant 25.0 × 23.0 × 5.0 cm lipomatous locking mesenteric mass was carefully dissected. It was encroaching onto the small bowel wall (Figure 3). After freeing the bowel from the mass, en bloc resection of the tumor and its capsule was subsequently performed. The patient had an uneventful postoperative course. Microscopic pathological examination resulted in a tumor composed of mature white adipose tissue with no evidence of nuclear atypia or mitosis (i.e., mesenteric lipoma).

## 3. Discussion

Lipoma is the most common mesenchymal and soft tissue tumor, has benign behavior and morphology, and is composed of mature white adipocytes with uniform nuclei resembling normal white fat.

Lipomas are usually subcutaneous and are found in the trunk and proximal extremities and less commonly on the hands, feet, and face. In rare cases, they may be found in the oral cavity, breast, pancreas, and intestines. There is an increased incidence of lipomas in patients with diabetes mellitus, hypercholesterolemia, and obesity, as found in our patient [3–5]. However, mesenteric lipoma is a rare tumor with less than 50 cases discussed in the English language literature [4, 5].

Mesenteric lipoma is mainly detected in adults between the ages of 40 and 60 years old [4], without any gender or ethnic preference, as reported before [6]. It is rare in children with the last case reported in April 2015. This lipoma was excised from a 2-year-old boy with 12.0 × 11.0 × 16.0 cm dimensions [7].

While it is usually asymptomatic due to their soft consistency, symptoms of small bowel obstruction mainly occur late and do not appear until the mass gets very large or is located near the intestinal lumen [4]. Some authors like Yang et al. reported unusual presentation such as an acute abdomen [8].

On the top of the differential diagnosis, come dermoid cyst, liposarcoma, lymphangioma, lipoblastoma, lymphangiolipoma, and neuroblastoma [3].

Mesenteric lipoma can be found incidentally during any abdominal imaging. Plain abdominal radiographs have no diagnostic value. Ultrasound may be used as a primary technique for the diagnosis of mesenteric lipoma as in our case; however, it is operator dependent and may misidentify mesenteric lipoma with usual mesenteric fat as seen with the case reported by Cha et al. [6]. Till now, computed tomography (CT) scan is the gold standard imaging technique with high detection rate of mesenteric lipoma where it can give specific and precise anatomical land marks [4, 6], which are useful for operative measures. MRI is also considered a very helpful diagnostic imaging with an advantage of differentiating lipomas from liposarcomas. This differentiation prevents from doing invasive diagnostic techniques such as biopsy before surgery [3, 9].

Treatment of mesenteric lipoma is mainly done by the complete resection of the tumor without affecting, if possible, the bowel loop. Laparoscopic surgery was accepted by many authors for the treatment of mesenteric lipomas [4]; however, despite its good results in case of small-sized lipoma, enlarging the incision is always needed as in the case reported by Kakiuchi et al. where the lipoma was of small size (9 × 10 cm), as compared to our lipoma size (25 × 23 × 5 cm) [10]. Exploratory laparotomy with en bloc resection of a giant mesenteric lipoma with or without intestinal resection remains the best option for treatment.

In conclusion, mesenteric lipoma is a rare entity. It should be considered in the differential diagnosis of chronic vague abdominal pain and distention and rarely does it present with an acute abdomen; it can reach a relatively great size, as in our case which is considered one of the largest mesenteric lipomas reported in the literature.
